# The narrowing gap in developed and developing country emission intensities reduces global trade’s carbon leakage

**DOI:** 10.1038/s41467-023-39449-7

**Published:** 2023-06-24

**Authors:** Jing Meng, Jingwen Huo, Zengkai Zhang, Yu Liu, Zhifu Mi, Dabo Guan, Kuishuang Feng

**Affiliations:** 1 The Bartlett School of Sustainable Construction, University College London, London, WC1E 6BT UK; 2 Department of Earth System Science, Ministry of Education Key Laboratory for Earth System Modeling, Institute for Global Change Studies, Tsinghua University, 100084 Beijing, China; 3 State Key Laboratory of Marine Environmental Science, College of the Environment and Ecology, Xiamen University, Xiamen, 361102 Fujian China; 4 College of Urban and Environmental Sciences, Peking University, 100871 Beijing, China; 5 Department of Geographical Sciences, University of Maryland, College Park, MD 20742 USA

**Keywords:** Climate-change mitigation, Climate-change policy, Carbon and energy

## Abstract

International trade affects CO_2_ emissions by redistributing production activities to places where the emission intensities are different from the place of consumption. This study focuses on the net emission change as the result of the narrowing gap in emission intensities between the exporter and importer. Here we show that the relocation of production activities from the global North (developed countries) to the global South (developing countries) in the early 2000s leads to an increase in global emissions due to the higher emission intensities in China and India. The related net emissions are about one-third of the total emissions embodied in the South-North trade. However, the narrowing emission intensities between South-North and the changing trade patterns results in declining net emissions in trade in the past decade. The convergence of emission intensities in the global South alleviates concerns that increasing South-South trade would lead to increased carbon leakage and carbon emissions. The mitigation opportunity to green the supply chain lies in sectors such as electricity, mineral products and chemical products, but calls for a universal assessment of emission intensities and concerted effort.

## Abstract (web summary)

International trade redistributes production activities to regions with varying emission intensities. This study finds that the convergence of emission intensities between the global South - North and changes in trade patterns have resulted in declining net emissions in trade in the past decade.

## Introduction

Current climate targets and policies are based on production-based emissions within jurisdictions, which measure emissions generated in the place where goods and services are produced. Trade allows for countries with lower emission intensities (i.e., carbon emissions per unit of production) to import goods from countries with higher emission intensities, which may lead to carbon leakage^1^. This has raised concerns that the separation of production and consumption may reduce the effectiveness of climate efforts^2,3^. In the 2000s, emissions from the production of globally traded goods and services (also known as “emissions embodied in trade” or “EET”) accounted for more than 20% of total global CO_2_ emissions^4,5^. The growing debate over EET lies in carbon redistribution, which has effects on regional mitigation responsibilities as well as impacts on global carbon emissions resulting from carbon intensity gaps across regions^6,7^.

Consumption-based accounting has, so far, been proposed by attributing the emissions embodied in imports to final consumers to adjust the mitigation responsibility^8,9^. However, this has been criticized for not taking technology differences or emission intensities into account^10,11^. Therefore, technology-adjusted consumption-based accounting within the multi-regional input-output framework has emerged to consider the technological differences between a country’s actual embodied emissions and hypothetical emissions if trade products are produced by an average world market^12,13^. Trade may help reduce world emissions if actual emissions are lower than hypothetical emissions due to more carbon-efficient production systems or specialization^10,14^. However, the point of the effect of international trade on global carbon emissions and how it can contribute to more carbon-efficient production worldwide is not yet fully understood. Furthermore, the pattern and volume of world trade are changing rapidly. The change in global trade patterns after the 2007–2008 financial crisis fueled concerns that emissions related to production would be passed across the global supply chain rather than being abated^1^.

This study aims to estimate the effects of the global trade on global carbon emissions (i.e., emissions from fossil fuel combustion) from 2004 to 2017, not only in the locations but also the amounts, and especially the role of technology differences. This study focuses on bilateral trade^11^ and the net emissions resulting from the gap in emissions intensity between imported and domestically produced products. The analysis of the changes in net emissions and the drivers behind them provides mitigation potential by closing the intensity gap.

## Results

### The different impacts of South-North trade and South-South trade

**Fig. 1:**
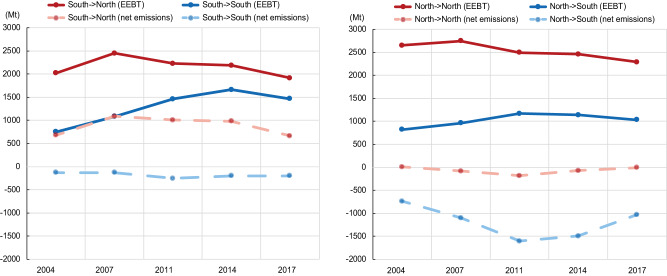
Change in emissions embodied in trade and net emissions resulting from South-South trade and South-North trade. South and North mean developing and developed countries, respectively. EEBT means the emissions embodied in bilateral trade.

The global South (i.e., developing countries) have been the dominant exporter since the early 2000s, while the global North (i.e., developed countries) have been the main importer. The total emissions embodied in South-North trade (i.e., trade from the global South to the global North) and South-South trade (i.e., trade between developing countries) followed similar trajectories from 2004 to 2017 (Fig. 1). From 2004 to 2007, escalating South-North trade contributed to the increase in global emissions, evidenced by the growth in net emissions, as the productions of many goods were outsourced from developed countries to China, where goods were produced at a much lower cost but in a more emissions-intensive way. The global financial crisis and subsequent responses from governments produced more erratic trade patterns from 2007 to 2011, making it difficult to discern longer-term structural shifts. The overall emissions embodied in bilateral trade (EEBT) related to South-North trade and South-South trade went in opposite directions during 2007–2014 and both decreased after 2014 because of a considerable drop in trade volume in 2015 and 2016.

One of the biggest concerns around EEBT relates to the potential contribution of trade to global emission increases because production activities tend to be shifted to regions with higher emission intensities and lower labor costs^9,15^. However, this study shows that global trade lowers total carbon emissions, evidenced by the net emissions (–227.7 Mt) in 2007. The net effects expanded to –568 Mt in 2017, while the total EEBT declined by 7.5%. The emissions reduction through global trade was largely contributed from the global North to the global South. In contrast, the South-North trade led to an increase in EEBT, while the net effect of the South-North trade declined from 2007 to 2017. The rising South-South trade showed a mitigation effect on the global EEBT with an increasing trend, but the contribution was still relatively small compared with the mitigation effects of the North-South trade.

**Fig. 2:**
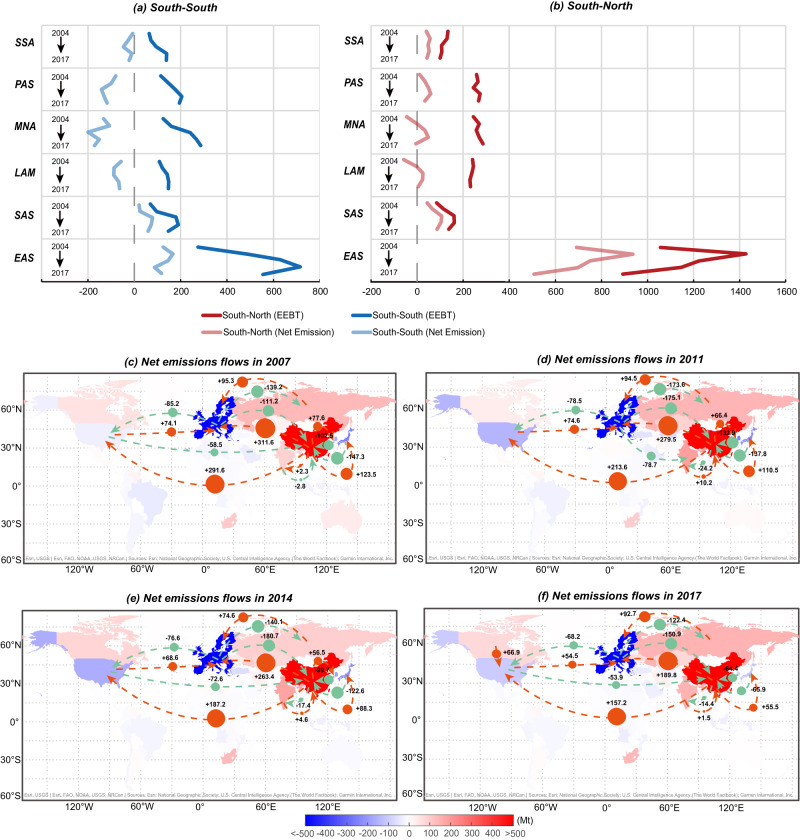
Net emissions embodied in exports. **a** Net emissions related to South-South trade; **b** Net emissions related to South-North trade; Net emissions flows in (**c**) 2007, (**d**) 2011, (**e**) 2014 and (**f**) 2017. EAS: China, SAS: India, LAM: Latin America and the Caribbean, MNA: the Middle East and North Africa, PAS: Pacific Developing regions in Asia and Pacific, SSA: sub-Saharan Africa. See details on the regional aggregation in the supplementary information. The regional color indicates the total net emissions embodied in exports. The red arrows mean positive net emissions embodied in bilateral emissions, while the green arrows mean negative net emissions. **c**–**f** Were created with the base map from ArcGIS, the attributions are on the map.

The biggest contributors to net emissions of trade were China and India (Fig. 2). The net emissions resulted from China’s exports to developed countries contributed 75–84% to the total net emissions from South-North trade, which climbed rapidly from 2004 to 2007 and then dropped until 2017. In contrast, the contribution of exports from India to developed countries increased from 5% in 2004 to 11% in 2011. The contributions from other regions are around 5%. In detail, the net emissions embodied in exports from China to the US declined from 291.6 Mt in 2007 to 157.2 Mt in 2017.

During 2004–2017, the net emissions embodied in exports from China and India to the global South contributed to increase in global emissions, despite the fact that South-South trade collectively lowered global emissions. In 2017, the total net emissions from exports to other developing countries in China and India were about a quarter of net emissions resulting from exports to developed countries. Even though emissions embodied in China’s exports to the global South also followed the inverse V curve, the peak was delayed until 2014. Specifically, in 2007, net emissions embodied in exports from China to India were calculated at +2.3 Mt, but by 2017, they had reversed direction to measure at −14.4 Mt (Fig. 2c and f).

### The narrowing emission intensity gap

**Fig. 3:**
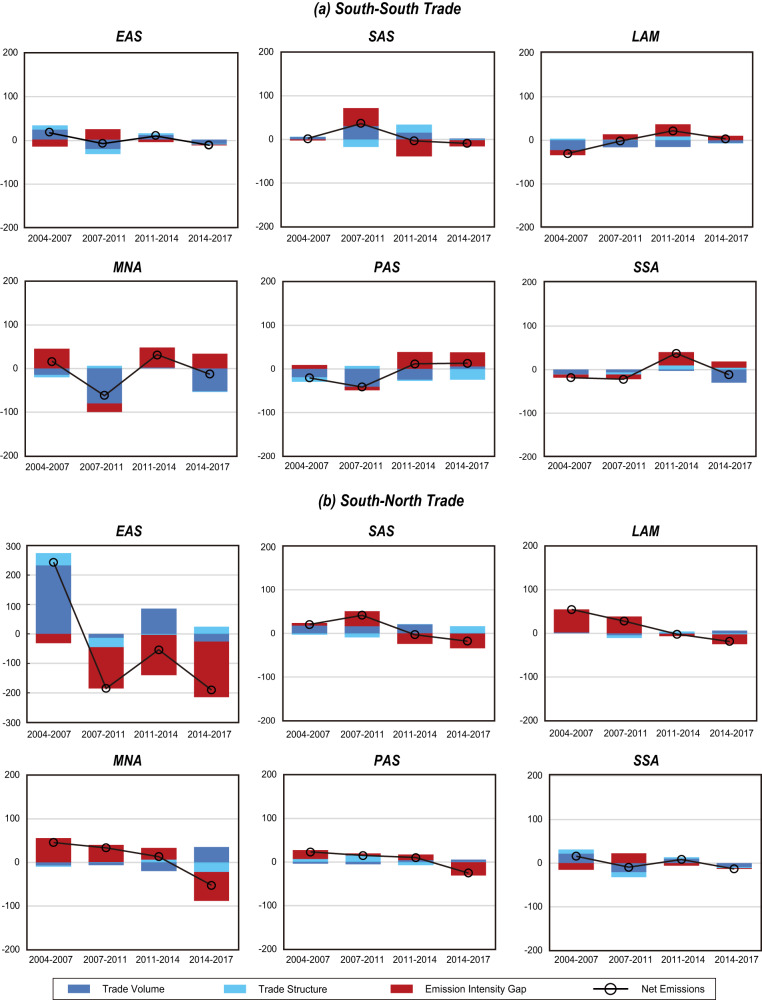
Decomposition of changes in net emissions related to exports from the six developing regions. **a** Net emissions related to South-South trade; **b** Net emissions related to South-North trade. Bars show the contributions of three indicators: trade volume, trade structure and emission intensity gap (see Methodology for details).

In both South-South and South-North trade, China’s exports collectively resulted in positive net emissions (Fig. 2). Nevertheless, the emissions intensity gaps have been shrinking over the study periods. By decomposing the change in net emissions, we found that emission intensity (i.e., carbon emissions per GDP) gaps were narrowing, except for the slight increase from 2014 to 2017 between China and other developing countries. Specifically, the decline in the emission intensity gap reduced 31.3 Mt of China’s exports to developed countries during 2004–2007 (Fig. 3b), while the effect escalated to 139.8, 136.4 and 189.0 Mt during 2007–2011, 2011–2014 and 2014–2017, respectively. Regarding the trade from China to other developing countries, the change in the emission intensity gap also contributed to offsetting the net emissions from 2004 to 2014. The gap did not change a lot from 2014 to 2017 because the emission intensities of traded products were quite similar among the global South, as evidenced by the declined contribution of the emission intensity gap among other regions.

India, as the second-largest country in terms of emissions embodied in export, has experienced fluctuations in its emission intensity over the past two decades. During this period, there was a wider emission intensity gap between India and its trade partners due to a 6% growth rate, from 1.07 kg/$ in 2007 to 1.13 kg/$ in 2011, resulting from the rapid increase in coal consumption (Table S5 in the Supplementary Information). The emission intensity gaps between India and its trade partners during 2007–2011 resulted in 40.6 Mt and 34.2 Mt of emissions embodied in India’s exports to the global South and global North, respectively. However, the emission intensity in India declined between 2011 and 2017 which narrowed the gaps with trade partners and decelerated net emissions during 2011–2014 and 2014–2017. Even though the emission intensity in India decreased by 19% after 2011, to 0.92 Kg/$ in 2017, it is still much higher than that of China (0.69 Kg/$). Overall, the emission intensities in large trading economies in the global South (e.g., China and India) were higher than those of their trade partners but declined more rapidly, leading to a gradual convergence of emission intensities with their major trading economies.

For the other four developing regions (LAM, Latin America and the Caribbean; MNA, the Middle East and North Africa; PAS, Pacific Developing regions in Asia and Pacific; and SSA, sub-Saharan Africa), the contributions of the emission intensity gap and trade volumes were fluctuating due to unclear trends in the trade volume and structure. However, there are some positive signals. The overall South-South trade led to negative net emissions; in other words, exports from those four regions to other developing regions contributed to a net emissions reduction. In contrast, even though exports to the global North resulted in positive net emissions, the effect of emission intensity gaps was declining.

### The heterogeneity of emission intensity gaps at the sectoral level

**Fig. 4:**
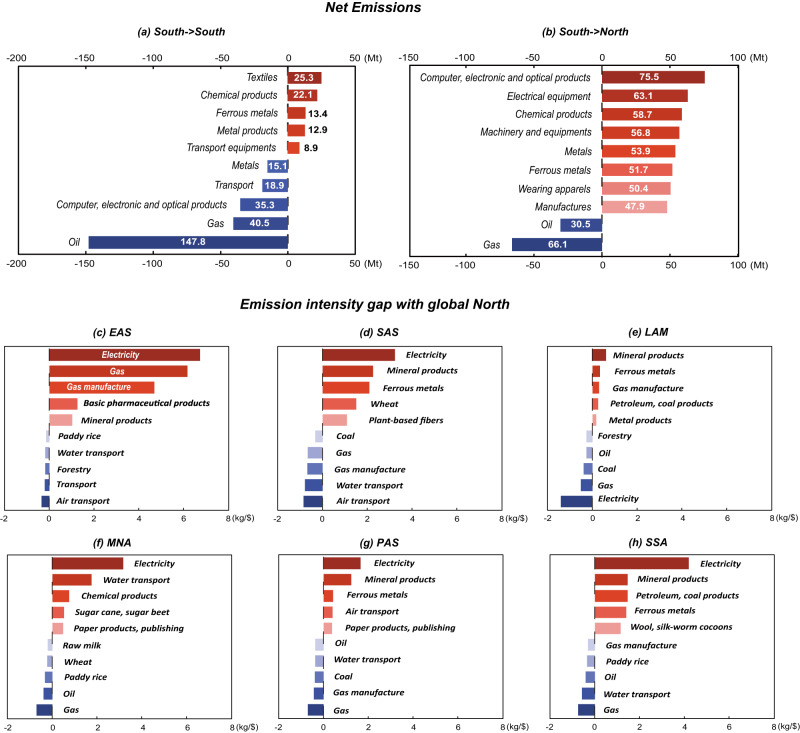
The sectoral contribution of net emissions related to trade export from the global South in 2017. **a** Export to the global South; **b** export to the global North. The difference in emission intensity (of traded products) from (**c**) EAS, (**d**) SAS, (**e**) LAM, (**f**) MNA, (**g**) PAS and (**h**) SSA compared with selected sectors with global North in 2017.

The net emissions related to global South-South trade were –197.5 Mt in 2017, mainly contributed by the sectors of oil (–147.8 Mt), gas (–40.5 Mt) and computer, electronic and optical products (–35.3 Mt) (Fig. 4). Specifically, they were dominated by exports of oil from the Middle East and North Africa (MNA) with an emission intensity of 0.11 kg/$ to China (EAS) with an emission intensity of 0.62 kg/$, and exports of gas from the Pacific Developing regions in Asia and Pacific (PAS) with an emission intensity of 0.07 kg/$ to China (EAS) with an emission intensity of 6.9 kg/$. Net emissions related to global South-North trade declined by 38.7% from 1085.0 Mt in 2007 to 665.6 Mt in 2017, mainly contributed by trade in emission-intense products, such as equipment and machinery, chemical products and metal products. Specifically, the contribution of the top eight sectors ranged from 47.9 Mt in other manufacturing (including furniture) to 75.5 Mt in computer, electronic and optical products. The emission intensity gap with global North for the traded products in the eight sectors also witnessed significant drops, except for ferrous metals where the decrease of emission intensity was only 6.7% from 2004 to 2017.

The emission intensity gap and mitigation potential differ at the sectoral level across regions, due to differences in resource endowments and regional relative advantages. The emission intensity gap between China and the global North lies in the electricity sector and gas sector, whose carbon emission intensities were 6.7 kg/$ (about three times that of the global North) and 6.2 kg/$ (about eight times) higher than those of the global North in 2017, respectively. China has the largest gap in the electricity sector among the six regions due to the dominant role of coal power plants. Similarly, Sub-Saharan Africa (SSA) also had a much higher emission intensity (a gap of 4.2 kg/$) in the electricity sector due to increased coal use^16^. In contrast, renewable energy generation in Latin America and the Caribbean (LAM) is relatively large^17^. Hydropower generation in Latin America accounted for more than 50% of the total power generation in 2016^18^. In Brazil, in particular, renewable energy sources accounted for an incredible 36.40% of the total energy matrix^19^. Therefore, the emission intensity of LAM’s power sector was lower than that of developed countries by 1.4 kg/$ (63.9% emission intensity of global North).

For the gas sector, in 2018, China’s gas import volume was about 125.4 billion cubic meters, with an increase of 31.7% from 2017. China’s import volume surpassed that of Japan, becoming the world’s largest gas importer, and its external dependence increased to 45.3%^20,21^. To reduce dependence on gas imports, China has facilitated unconventional natural gas (e.g., shale gas and coal-based synthetic natural gas (SNG)) as alternative natural gas suppliers^22,23^. The SNG industry is coal-based and results in higher carbon emission intensity in this industry^24^.

In India (SAS), the observed emission intensity gaps were electricity, mineral products and ferrous metals. The mining sector accounts for about 2.5% of India’s GDP due to considerable advantages in the production costs of steel and alumina^25^. Therefore, India’s mining industry was expanding very fast, accompanied by the related CO_2_ emissions^26^. India is one of the largest iron ore and bauxite producers in the world and its carbon emission intensity is higher than that of developed countries by 2.2 kg/$. Therefore, this industry is a large contributor to net emissions related to exports from India.

## Discussion

Trade has allowed countries with higher emission intensities to export goods to countries with lower emission intensities, which may lead to an increase in global carbon emissions. Even though the impact of the emission intensities gap is narrowing, trade between the global South and global North contributes to increases in global emissions, especially in emission-intensive sectors such as ferrous metals, mineral products and chemical products. This reveals mitigation potential in these industries and calls for efforts to reduce the gap.

**Fig. 5:**
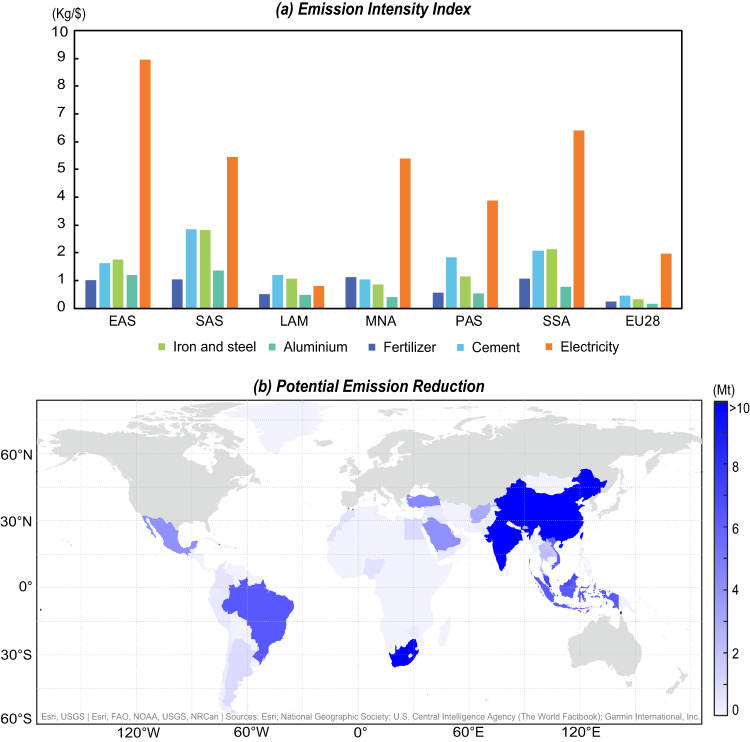
Potential impact on emission reduction embodied in exports from global South countries. **a** The sectoral emission intensity of selected product exports in 2019; **b** The potential for emissions reduction in each global South country in 2019 if the sectoral emission intensity of each country matched the emission intensity level of the European Union. **b** Was created with the base map from ArcGIS, the attributions are on the map.

Generally, the emission intensity of these high carbon-intensive sectors in the six global South regions is larger than in the EU. Especially in the electricity sector, the emission intensity in Sub-Saharan Africa (SSA) is 6.4 kg/$, which is about three times that in the EU (2.0 kg/$). Because the export of iron and aluminum was relatively large in 2019 for the global South region (iron and steel: $112.5 billion; aluminum: $59.4 billion), emission reductions for these products will contribute significantly when the emission intensity in the global South region declines to the level in the EU (Fig. 5). For example, China will decrease 56.7 Mt emissions embodied in the export of iron and steel, and 27 Mt emissions embodied in the export of aluminum in 2019.

Despite the positive impact on carbon emissions, more attention should also be paid to potential economic risks in the global South to avoid new global dividing lines resulting from different export structures^27^. Therefore, the global North can provide more technical and financial support to help the global South achieve their Nationally Determined Contributions (NDCs) and sustainable development in the economic and social dimensions. For example, the revenue from CBAM can be used to invest in required infrastructure in the global South to help developing countries achieve a just green transition^28,29^. For energy-intensive commodities such as cement and steel that are not easy to decarbonize, the EU can prevent negative chain reactions in developing countries by investing resources and technologies to improve the efficiency of industrial processes and by providing funds for renewable energy projects^30^. At the same time, the EU could also use some of the revenue from CBAM to help developing countries adopt cleaner technologies^31^.

However, the pollution haven hypothesis reveals that lax environmental enforcement in developing countries attracts investment in emission-intensive industries from developed countries, especially in the context of increasing numbers of countries committing to carbon neutrality before 2050. Even though 192 countries (with 96% of global emissions) have submitted ambitious mitigation targets, and 83.5% of global South countries have submitted NDCs^32,33^, it will challenge global mitigation efforts by reshaping the global supply chain and emission hotspots. Currently, the shrinking technological gap between the global South and North is mainly influenced by China, which has promised to be net zero before 2060. Most of the least developed countries (i.e., low-income countries confronting severe structural impediments to sustainable development) are still struggling with poverty and economic growth. The global North should engage with the global South in designing mitigation targets and pathways, as well as providing essential assistance. Dealing with the COVID-19 pandemic has proven the power of international collaboration, which is also key to climate change mitigation.

Even though this study focuses on the trend from 2004 to 2017, the narrowing gap still stands during 2017–2019, because of the rapid decline in coal share and emission intensity in the global South (Table S5). Future studies are expected to be extended to Non-CO_2_ to harness the power of trade in collaborative climate change mitigation.

## Methods

### Data source

The economic input-output data and sectoral CO_2_ emissions are from version 11 of the Global Trade Analysis Project (GTAP) database^34^, which includes 141 regions and 65 sectors in 2004, 2007, 2011, 2014 ad 2017. The 141 regions are divided into 10 groups based on geography and economic levels: China, India, Middle East and North Africa, Latin America and the Caribbean, Sub-Saharan Africa, and Other Asia and Pacific; and other (mostly developed) regions of US-North America, Western Europe, Eastern Europe and the former Soviet Union, and developed Asia-Pacific regions (see supplementary Tables S1 and S2). The carbon emissions in this study cover only emissions from fossil fuel combustion.

### Emissions embodied in bilateral trade

Originally developed by Leontief^35^, environmental input-output analyses have been widely used to illustrate the economy-wide environmental repercussions triggered by economic activities. Both the EEBT and Multi-Regional Input-Output models (MRIO) methods can be used to calculate the emissions embodied in trade. MRIO links the producer where the emissions are originally produced and the final consumers of the products regardless of how many times they have been traded across countries, and includes the intermediate consumption endogenously^36^. In contrast, EEBT focuses on embodied emissions in the bilateral trade of both intermediate and final consumption by tracing the domestic supply chain^5^. Here we analyzed the emissions embodied in bilateral trade (EEBT), which captures emissions embodied in both intermediate and final products, which is more suitable to identify the effect of trade compared with MRIO^1^. For example, Meng et al.^1^ used EEBT to calculate the CO_2_ emissions embodied in South-South trade and the contribution of the complex supply chains to the emissions production of global south. Huo et al (2021) used EEBT to assess the change in emissions embodied in service trade and quantified the contribution of socioeconomic drivers.

We used global MRIO tables for 2004, 2007, 2011, and 2014 from GTAP v10 and the table for 2017 from v11. To be consistent, the table in 2017 has been aggregated into 141 regions and 65 sectors. The global MRIO framework begins with the accounting balance of monetary flows between industrial sectors and regions:

1 $$\left(\begin{array}{c}{x}^{1}\\ {x}^{2}\\ \vdots \\ {x}^{m}\end{array}\right)=\left(\begin{array}{cccc}{Z}^{11} & {Z}^{12} & \cdots & {Z}^{1m}\\ {Z}^{21} & {Z}^{22} & \cdots & {Z}^{2m}\\ \vdots & \vdots & \ddots & \vdots \\ {Z}^{m1} & {Z}^{m2} & \cdots & {Z}^{{mm}}\end{array}\right)+\left(\begin{array}{c}{\sum }_{s}\, {y}^{1s}\\ {\sum }_{s}\, {y}^{2s}\\ \vdots \\ {\sum }_{s}\, {y}^{{ms}}\end{array}\right)$$

where x^r^ is a vector for sectoral total outputs in region r; ${Z}^{rs}$ represents the industry requirements from region *r* to produce output in region *s*; ${y}^{rs}$ is the final demand (household, government and investment) supply from region *r* to s; and *m* indicates the total number of regions, which was 141 in this study.

For each region, the monetary balance is:

2 $${{{{{{\boldsymbol{x}}}}}}}^{r}={{{{{{\boldsymbol{Z}}}}}}}^{r}+{{{{{{\boldsymbol{y}}}}}}}^{r}+\mathop{\sum }\limits_{s}{{{{{{\boldsymbol{e}}}}}}}^{{rs}}-\mathop{\sum }\limits_{s}{{{{{{\boldsymbol{e}}}}}}}^{{sr}}$$

where e^rs^ (r ≠ s) is the exports from region *r* to *s*. In the framework of EEBT, imports are removed from intermediate (Z^r^) and final products (y^r^) to focus on domestic production only:

3 $${{{{{{\boldsymbol{x}}}}}}}^{r}={{{{{{\boldsymbol{Z}}}}}}}^{{rr}}+{{{{{{\boldsymbol{y}}}}}}}^{{rr}}+\mathop{\sum }\limits_{s}{{{{{{\boldsymbol{e}}}}}}}^{{rs}}$$

The total direct and indirect emissions in region *r* related to exports to region *s* are:

4 $${{{{{{\boldsymbol{T}}}}}}}^{{rs}}={{{{{{\boldsymbol{F}}}}}}}^{r}{({{{{{\boldsymbol{I}}}}}}-{{{{{{\boldsymbol{A}}}}}}}^{{rr}})}^{-1}{{{{{{\boldsymbol{e}}}}}}}^{{rs}}$$

where **F**^r^ is the direct emission intensity in region *r*, which is obtained by dividing the CO_2_ emissions in each sector by the corresponding output. While ${({{{{{\boldsymbol{I}}}}}}-{{{{{{\boldsymbol{A}}}}}}}^{r})}^{-1}$ is the Leontief inverse matrix which captures both direct and indirect emissions to produce one unit of the final product, ${({{{{{\boldsymbol{I}}}}}}-{{{{{{\boldsymbol{A}}}}}}}^{{rr}})}^{-1}$ captures the domestic supply chain in region *r*. ${{{{{{\boldsymbol{h}}}}}}}^{r}={{{{{{\boldsymbol{F}}}}}}}^{r}{({{{{{\boldsymbol{I}}}}}}-{{{{{{\boldsymbol{A}}}}}}}^{{rr}})}^{-1}$ is the embodied emission intensity which captures direct and indirect carbon emissions along the supply chain to produce a unit of product or service.

### Emissions displacement and net emissions

As we argue in the introduction, the traditional concept of EEBT does not separate emissions displacement and net emissions. The intuition to separate them is because the effects and policy implications are different for the two types of emissions. The basic idea of emissions displacement is to estimate the emissions avoided by importers to satisfy the demand for production or consumption, so the emissions displacement is:

5 $$T{D}_{i}^{{rs}}={e}_{i}^{{rs}}{h}_{i}^{s}$$

where ${T}_{i}^{{rs}}$ is the emissions displaced from region r to s due to exports from sector *i* in region *r* to region *s*. ${e}_{i}^{{rs}}$ means the exports from sector *i* in region *r* to region *s*. ${h}_{i}^{s}$ is the embodied carbon emissions to produce unit products or services in sector *i* in region *s*.

The net emissions mean the total emissions change due to the difference between the exporter and importer, which can be calculated as:

6 $$T{I}_{i}^{{rs}}={e}_{i}^{{rs}}({h}_{i}^{r}-{h}_{i}^{s})$$

If the emission intensity in sector *i* in region *r* (${h}_{i}^{r}$) is higher than that in region s (${h}_{i}^{s}$), the $T{I}_{i}^{{rs}}$ will be positive, which means that traded products in sector *i* between region r and s increase global carbon emissions. The $T{I}_{i}^{{rs}}$ will be negative if ${h}_{i}^{r}$ is lower than ${h}_{i}^{s}$, which means the trade contributes to a reduction in carbon emissions.

### Decomposition analysis

Decomposition analysis methods have been used extensively to assess the contribution of socioeconomic drivers to changes in carbon emissions^37–39^. The two most common decomposition methods are Index Decomposition Analysis (IDA) and Structural Decomposition Analysis (SDA)^40^. IDA is more widely used, as it has relatively low data requirements and is flexible^41,42^. Many studies have used IDA to provide policy-relevant insights, for instance by identifying the driving forces of energy consumption in developing economies^43,44^ and changes in CO_2_ emissions^45–47^. The IDA compares a set of indices between the base and final year of a given period and decomposes these dependent variables into various independent determinants to explore the effects of the indices on the trend of emissions over that period^40^.

In our analysis, we divided the change in net emissions into three constituent parts: trade volume, trade structure, and the emission intensity gap effect.

The total net CO_2_ emissions of producing the products exported from region *r* to region *s* can be decomposed as follows:

7 $${{{{{\bf{T}}}}}}{{{{{{\bf{I}}}}}}}^{rs}=\mathop{\sum}\limits_{i}{e}_{i}^{rs}({h}_{i}^{r}-{h}_{i}^{s})=\mathop{\sum}\limits_{i}{e}^{rs}\frac{{e}_{i}^{rs}}{{e}^{rs}}({h}_{i}^{r}-{h}_{i}^{s})=\mathop{\sum}\limits_{i}{E}^{rs}{S}_{i}^{rs}{H}_{i}^{rs}$$

where ${{{{{\boldsymbol{T}}}}}}{{{{{{\boldsymbol{I}}}}}}}^{{rs}}$ is the net emissions generated from producing the total exports from region *r* to region *s*; ${E}^{rs}$ represents the total export volume from region *r* to region *s*; ${S}_{i}^{rs}$ represents the export share of sector *i* from region *r* to region *s*; ${H}_{i}^{rs}$ represents the emission intensity gap of sector *i* between in region *r* and region *s*.

Thus, the change in the net emissions between two points in time (indicated by the subscripts 0 and 1) can be expressed as $\varDelta {{{{{\boldsymbol{T}}}}}}{{{{{{{\boldsymbol{I}}}}}}}_{a}}^{{rs}}={{{{{\boldsymbol{T}}}}}}{{{{{{{\boldsymbol{I}}}}}}}_{1}}^{{rs}}-{{{{{\boldsymbol{T}}}}}}{{{{{{{\boldsymbol{I}}}}}}}_{0}}^{{rs}}$. Because the decomposition is not unique, when the number of factors is m, the number of all possible equivalent decompositions is equal to $m\, !$. To resolve the non-uniqueness problem, we apply an established method using the average of the termed polar decompositions as an approximation of the average of all $m\, !$ equivalent decomposition forms^48^. The two polar decompositions ($\varDelta {{{{{\bf{T}}}}}}{{{{{{\bf{I}}}}}}}_{a}^{{{{{{\rm{rs}}}}}}}$ and $\varDelta {{{{{\bf{T}}}}}}{{{{{{\bf{I}}}}}}}_{b}^{{{{{{\rm{rs}}}}}}}$) are as follows:

8 $$\varDelta {{{{{\bf{T}}}}}}{{{{{{{\bf{I}}}}}}}_{a}}^{rs}=	\mathop{\sum}\limits_{i}(\varDelta {E}^{rs}){S}_{i0}^{rs}{H}_{i0}^{rs}+\mathop{\sum}\limits_{i}{E}_{1}^{rs}(\varDelta {S}_{i}^{rs}){H}_{i0}^{rs} \\ 	+\mathop{\sum}\limits_{i}{E}_{1}^{rs}{S}_{i1}^{rs}(\varDelta {H}_{i}^{rs})=\varDelta {{{{{{\bf{E}}}}}}}_{a}+\varDelta {{{{{{\bf{S}}}}}}}_{a}+\varDelta {{{{{{\bf{H}}}}}}}_{a}$$

9 $$\varDelta {{{{{\bf{T}}}}}}{{{{{{{\bf{I}}}}}}}_{b}}^{rs}=	\mathop{\sum}\limits_{i}(\varDelta {E}_{i}^{rs}){S}_{i1}^{rs}{H}_{i1}^{rs}+\mathop{\sum}\limits_{i}{E}_{i0}^{rs}(\varDelta {S}_{i}^{rs}){H}_{i1}^{rs} \\ 	+\mathop{\sum}\limits_{i}{E}_{i0}^{rs}{S}_{i0}^{rs}(\varDelta {H}_{i}^{rs})=\varDelta {{{{{{\bf{E}}}}}}}_{b}+\varDelta {{{{{{\bf{S}}}}}}}_{b}+\varDelta {{{{{{\bf{H}}}}}}}_{b}$$

The average of the polar decomposition is expressed as follows:

10 $$\varDelta {{{{{\bf{T}}}}}}{{{{{{\bf{I}}}}}}}^{rs}=	\frac{1}{2}[\varDelta {{{{{\bf{T}}}}}}{{{{{{\bf{I}}}}}}}_{a}^{rs}+\varDelta {{{{{\bf{T}}}}}}{{{{{{\bf{I}}}}}}}_{b}^{rs}]=\frac{1}{2}(\varDelta {{{{{{\bf{E}}}}}}}_{a}+\varDelta {{{{{{\bf{E}}}}}}}_{b})+\frac{1}{2}(\varDelta {{{{{{\bf{S}}}}}}}_{a}+\varDelta {{{{{{\bf{S}}}}}}}_{b}) \\ 	+\frac{1}{2}(\varDelta {{{{{{\bf{H}}}}}}}_{a}+\varDelta {{{{{{\bf{H}}}}}}}_{b})=\varDelta {{{{{\bf{E}}}}}}+\varDelta {{{{{\bf{S}}}}}}+\varDelta {{{{{\bf{H}}}}}}$$

where $\varDelta {{{{{\bf{{T}}}}}}}{{{{{{\bf{I}}}}}}}^{{{{{{\rm{rs}}}}}}}$ is the change in net emission transfers between two points in time, which in this study corresponds to 2004–2007, 2007–2011, 2011–2014 and 2014–2017.$\varDelta {{{{{\boldsymbol{E}}}}}}$, $\varDelta {{{{{\boldsymbol{S}}}}}}$ and $\varDelta {{{{{\boldsymbol{H}}}}}}$ refer to the trade volume effect, trade structure effect and emission intensity gap effect, respectively.

## Supplementary information

Supplementary Information

## Data Availability

The multi-regional input-output tables and CO_2_ emissions are from GTAP database, version 11 (https://www.gtap.agecon.purdue.edu/databases) upon licence. The data generated in this study have been deposited in figureshare (10.6084/m9.figshare.22777265). Please contact the corresponding authors for more details.

## Code availability

Requests for code developed in MATLAB to process and analyze the primary data collected in this study will be made available upon request.
