# Supplementary material for: The narrowing gap in developed and developing country emission intensities reduces global trade’s carbon leakage
Source: Nat Commun. 2023 Jun 24;14:3775. doi: 10.1038/s41467-023-39449-7 (PMC10290715; doi:10.1038/s41467-023-39449-7)
Supplement: Supplementary file 1 — Supplementary Information [file 41467_2023_39449_MOESM1_ESM.pdf]

Supporting Information

The narrowing gap in developed and developing country emission intensities reduces global trade's carbon leakage

Meng et al

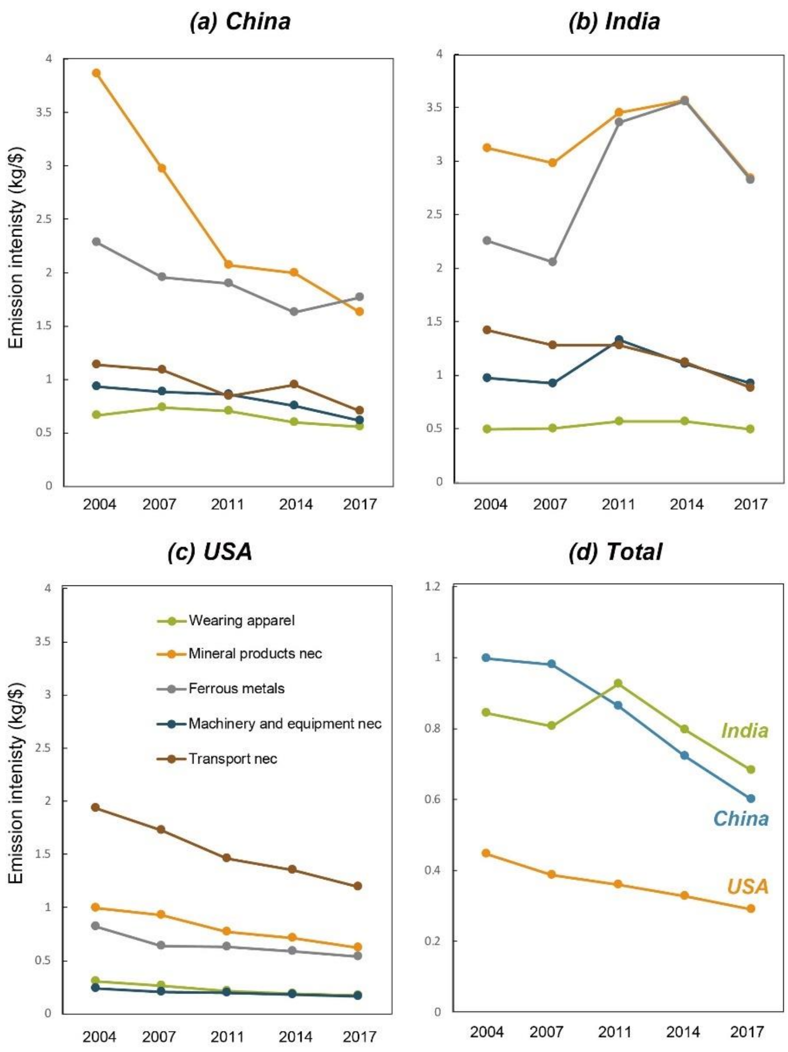

Figure. S1. Annual changes in both national total embodied emission intensities (d) and embodied emission intensities in selected types of products exported by China (a), India (b) and USA (c).

Table S1. Definition of regions.

Number	Code	Description	Member Countries
1	AUS	Australia	Australia, Cocos (Keeling) Islands, Heard Island and McDonald Islands, Norfolk Island
2	NZL	New Zealand	New Zealand
3	XOC	Rest of Oceania	American Samoa, Cook Islands, Fiji, Micronesia Federated States of, Guam, Kiribati, Marshall Islands, Northern Mariana Islands, New Caledonia, Niue, Nauru, Palau, Papua New Guinea, French Polynesia, Solomon Islands, Tokelau, Tonga, Tuvalu, Vanuatu, Wallis and Futuna, Samoa, Pitcairn, United States Minor Outlying Islands
4	CHN	China	China
5	HKG	Hong Kong	Hong Kong
6	JPN	Japan	Japan
7	KOR	Korea Republic of	Korea Republic of
8	MNG	Mongolia	Mongolia
9	TWN	Taiwan	Taiwan
10	XEA	Rest of East Asia	,Korea, Democratic People’s Republic of
11	BRN	Brunei Darussalam	Brunei Darussalam
12	KHM	Cambodia	Cambodia
13	IDN	Indonesia	Indonesia
14	LAO	Lao Peoples Democratic Republic	Lao Peoples Democratic Republic
15	MYS	Malaysia	Malaysia
16	PHL	Philippines	Philippines
17	SGP	Singapore	Singapore
18	THA	Thailand	Thailand
19	VNM	Viet Nam	Viet Nam
20	XSE	Rest of Southeast Asia	Myanmar, Timor-Leste
21	BGD	Bangladesh	Bangladesh
22	IND	India	India
23	NPL	Nepal	Nepal
24	PAK	Pakistan	Pakistan
25	LKA	Sri Lanka	Sri Lanka
26	XSA	Rest of South Asia	Afghanistan, Bhutan, Maldives
27	CAN	Canada	Canada
28	USA	United States of America	United States of America
29	MEX	Mexico	Mexico
30	XNA	Rest of North America	Bermuda, Greenland, Saint Pierre and

			Miquelon
31	ARG	Argentina	Argentina
32	BOL	Bolivia	Bolivia
33	BRA	Brazil	Brazil
34	CHL	Chile	Chile
35	COL	Colombia	Colombia
36	ECU	Ecuador	Ecuador
37	PRY	Paraguay	Paraguay
38	PER	Peru	Peru
39	URY	Uruguay	Uruguay
40	VEN	Venezuela	Venezuela
41	XSM	Rest of South America	Falkland Islands (Malvinas), French Guiana, Guyana, Suriname, South Georgia and the South Sandwich Islands
42	CRI	Costa Rica	Costa Rica
43	GTM	Guatemala	Guatemala
44	HND	Honduras	Honduras
45	NIC	Nicaragua	Nicaragua
46	PAN	Panama	Panama
47	SLV	El Salvador	El Salvador
48	XCA	Rest of Central America	Belize
49	DOM	Dominican Republic	Dominican Republic
50	JAM	Jamaica	Jamaica
51	PRI	Puerto Rico	Puerto Rico
52	TTO	Trinidad and Tobago	Trinidad and Tobago
53	XCB	Caribbean	Aruba, Anguilla, Netherlands Antilles (Curacao, Bonaire, Sint Eustatius and Saba), Antigua & Barbuda, Bahamas, Barbados, Cuba, Cayman Islands, Dominica, Grenada, Haiti, Saint Kitts and Nevis, Saint Lucia, Montserrat, Turks and Caicos Islands, Saint Vincent and the Grenadines, Virgin Islands British, Virgin Islands U.S.
54	AUT	Austria	Austria
55	BEL	Belgium	Belgium
56	BGR	Bulgaria	Bulgaria
57	HRV	Croatia	Croatia
58	CYP	Cyprus	Cyprus
59	CZE	Czech Republic	Czech Republic
60	DNK	Denmark	Denmark
61	EST	Estonia	Estonia
62	FIN	Finland	Finland, Aland Islands
63	FRA	France	France, Guadeloupe, Martinique, Reunion
64	DEU	Germany	Germany

65	GRC	Greece	Greece
66	HUN	Hungary	Hungary
67	IRL	Ireland	Ireland
68	ITA	Italy	Italy
69	LVA	Latvia	Latvia
70	LTU	Lithuania	Lithuania
71	LUX	Luxembourg	Luxembourg
72	MLT	Malta	Malta
73	NLD	Netherlands	Netherlands
74	POL	Poland	Poland
75	PRT	Portugal	Portugal
76	ROU	Romania	Romania
77	SVK	Slovakia	Slovakia
78	SVN	Slovenia	Slovenia
79	ESP	Spain	Spain
80	SWE	Sweden	Sweden
81	GBR	United Kingdom	United Kingdom
82	CHE	Switzerland	Switzerland
83	NOR	Norway	Norway, Svalbard and Jan Mayen
84	XEF	Rest of EFTA	Iceland, Liechtenstein
85	ALB	Albania	Albania
86	BLR	Belarus	Belarus
87	RUS	Russian Federation	Russian Federation
88	UKR	Ukraine	Ukraine
89	XEE	Rest of Eastern Europe	Moldova Republic of
90	XER	Rest of Europe	Andorra, Bosnia and Herzegovina, Faroe Islands, Gibraltar, Monaco, Macedonia the former Yugoslav<br>Republic of San Marino, Serbia, Guernsey, Isle of Man, Jersey, Montenegro, Holy See (Vatican City State)
91	KAZ	Kazakhstan	Kazakhstan
92	KGZ	Kyrgyzstan	Kyrgyzstan
93	TJK	Tajikistan	Tajikistan
94	XSU	Rest of Former Soviet Union	Turkmenistan, Uzbekistan
95	ARM	Armenia	Armenia
96	AZE	Azerbaijan	Azerbaijan
97	GEO	Georgia	Georgia
98	BHR	Bahrain	Bahrain
99	IRN	Iran Islamic Republic of	Iran Islamic Republic of
100	ISR	Israel	Israel
101	JOR	Jordan	Jordan
102	KWT	Kuwait	Kuwait

103	OMN	Oman	Oman
104	QAT	Qatar	Qatar
105	SAU	Saudi Arabia	Saudi Arabia
106	TUR	Turkey	Turkey
107	ARE	United Arab Emirates	United Arab Emirates
108	XWS	Rest of Western Asia	Iraq, Lebanon, Palestinian Territory Occupied, Syrian Arab Republic, Yemen
109	EGY	Egypt	Egypt
110	MAR	Morocco	Morocco
111	TUN	Tunisia	Tunisia
112	XNF	Rest of North Africa	Algeria, Libya, Western Sahara
113	BEN	Benin	Benin
114	BFA	Burkina Faso	Burkina Faso
115	CMR	Cameroon	Cameroon
116	CIV	Cote dIvoire	Cote dIvoire
117	GHA	Ghana	Ghana
118	GIN	Guinea	Guinea
119	NGA	Nigeria	Nigeria
120	SEN	Senegal	Senegal
121	TGO	Togo	Togo
122	XWF	Rest of Western Africa	Cape Verde, Gambia, Guinea-Bissau, Liberia, Mali, Mauritania, Niger, Saint Helena, Ascension and Tristan da Cunha, Sierra Leone
123	XCF	Central Africa	Central African Republic, Congo, Gabon, Equatorial Guinea, Sao Tome and Principe, Chad
124	XAC	South Central Africa	Angola, Congo, Democratic Republic of the
125	ETH	Ethiopia	Ethiopia
126	KEN	Kenya	Kenya
127	MDG	Madagascar	Madagascar
128	MWI	Malawi	Malawi
129	MUS	Mauritius	Mauritius
130	MOZ	Mozambique	Mozambique
131	RWA	Rwanda	Rwanda
132	TZA	Tanzania United Republic of	Tanzania United Republic of
133	UGA	Uganda	Uganda
134	ZMB	Zambia	Zambia
135	ZWE	Zimbabwe	Zimbabwe
136	XEC	Rest of Eastern Africa	Burundi, Comoros, Djibouti, Eritrea, Mayotte, Sudan (includes South Sudan), Somalia, Seychelles
137	BWA	Botswana	Botswana

138	NAM	Namibia	Namibia
139	ZAF	South Africa	South Africa
140	XSC	Rest of South African Customs Union	Lesotho, Eswatini (Swaziland prior to 2018)
141	XTW	Rest of the World	Antarctica, French Southern Territories, Bouvet Island, British Indian Ocean Territory

Table S2. Abbreviation and concordance of world regions.

World region	Abbreviation	GTAP regions
China	EAS	4
India	SAS	22
Economies in Transition (Eastern Europe and former Soviet Union)	EIT	56,57,59,61,66,69,70,74,76,77,78,87,88,89,90,91,92,93,94,95,96,97
Latin America and Caribbean	LAM	29,30,31,32,33,34,35,36,37,38,39,40,41,42,43,44,45,46,47,48,49,50,51,52,53
Middle East and North Africa	MNA	98,99,100,101,102,103,104,105,106,107,108,109,110,111,112
North America (USA, Canada)	NAM	27,28
Pacific Organization for Economic Co-operation and Development 1990	POECD	1,2,5,6,7,9,17
Pacific Developing regions in Asia and Pacific	PAS	3,8,10,11,12,13,14,15,16,18,19,20,21,23,24,25,26
sub-Saharan Africa	SSA	113,114,115,116,117,118,119,120,121,122,123,124,125,126,127,128,129,130,131,132,1333,134,135,136,137,138,139,140,141
Western Europe	WEU	54,55,58,60,62,63,64,65,67,68,71,72,73,75,79,80,81,82,83,84,85

Table S3. Definition of sectors.

**Sector**	
1	Paddy rice
2	Wheat
3	Cereal grains nec
4	Vegetables, fruit, nuts
5	Oil seeds
6	Sugar cane, sugar beet
7	Plant-based fibers
8	Crops nec
9	Cattle, sheep and goats, horses
10	Animal products nec
11	Raw milk
12	Wool, silk-worm cocoons
13	Forestry
14	Fishing
15	Coal
16	Oil
17	Gas
18	Other Extraction
19	Meat: cattle, sheep, goats, horse
20	Meat products nec
21	Vegetable oils and fats
22	Dairy products
23	Processed rice
24	Sugar
25	Food products nec
26	Beverages and tobacco products
27	Textiles
28	Wearing apparel
29	Leather products
30	Wood products
31	Paper products, publishing
32	Petroleum, coal products
33	Chemical products
34	Basic pharmaceutical products
35	Rubber and plastic products
36	Mineral products nec
37	Ferrous metals
38	Metals nec
39	Metal products
40	Computer, electronic and optical products

41	Electrical equipment
42	Machinery and equipment nec
43	Motor vehicles and parts
44	Transport equipment nec
45	Manufactures nec
46	Electricity
47	Gas manufacture, distribution
48	Water
49	Construction
50	Trade
51	Accommodation, Food and service activities
52	Transport nec
53	Water transport
54	Air transport
55	Warehousing and support activities
56	Communication
57	Financial services nec
58	Insurance
59	Real estate activities
60	Business services nec
61	Recreational and other services
62	Public Administration and defense
63	Education
64	Human health and social work activities
65	Dwellings

Table S4. The share of each contributor in changes in net emissions related to exports from the six developing regions.

South-South				
**EAS**	**2004-2007**	**2007-2011**	**2011-2014**	**2014-2017**
Trade Volume (%)	192.3	-340.8	-82.9	40.4
Trade Structure (%)	-33.3	-20.9	34.3	39.5
Emission intensity Gap (%)	-59.0	461.7	148.6	20.1
Total net emissions change (Mt)	39.87	-21.41	-57.20	29.66
**SAS**	**2004-2007**	**2007-2011**	**2011-2014**	**2014-2017**
Trade Volume (%)	142.0	57.1	-301.4	-15.7
Trade Structure (%)	22.0	-32.3	-360.8	11.8
Emission intensity Gap (%)	-64.1	75.3	762.2	103.9
Total net emissions change (Mt)	3.86	54.01	-5.13	-13.86
**LAM**	**2004-2007**	**2007-2011**	**2011-2014**	**2014-2017**
Trade Volume (%)	75.0	957.1	-68.6	-195.3
Trade Structure (%)	-13.3	-120.1	41.5	-13.5
Emission intensity Gap (%)	38.3	-737.0	127.1	308.8
Total net emissions change (Mt)	-30.08	-1.63	21.83	3.36
**MNA**	**2004-2007**	**2007-2011**	**2011-2014**	**2014-2017**
Trade Volume (%)	-56.6	86.4	4.2	269.6
Trade Structure (%)	-20.4	-7.0	0.6	6.0
Emission intensity Gap (%)	177.1	20.6	95.2	-175.6
Total net emissions change (Mt)	25.79	-92.70	47.87	-19.40
**PAS**	**2004-2007**	**2007-2011**	**2011-2014**	**2014-2017**
Trade Volume (%)	95.8	98.3	-212.9	45.1
Trade Structure (%)	49.0	-17.5	-26.3	-191.3
Emission intensity Gap (%)	-44.7	19.2	339.2	246.2
Total net emissions change (Mt)	-20.45	-41.40	11.45	13.00
**SSA**	**2004-2007**	**2007-2011**	**2011-2014**	**2014-2017**
Trade Volume	57.9	27.9	-8.1	256.1
Trade Structure	4.5	21.7	25.7	-34.5
Emission intensity Gap	37.7	50.5	82.4	-121.6
Total net emissions change (Mt)	-18.34	-21.95	37.36	-11.80
**South-North (%)**				
**EAS**	**2004-2007**	**2007-2011**	**2011-2014**	**2014-2017**
Trade Volume	95.7	7.3	-158.9	13.7
Trade Structure	17.2	17.0	5.9	-13.0
Emission intensity Gap	-12.9	75.7	252.9	99.3
Total net emissions change (Mt)	243.53	-184.81	-53.94	-190.27
**SAS**	**2004-2007**	**2007-2011**	**2011-2014**	**2014-2017**
Trade Volume	87.0	39.9	-739.2	1.4
Trade Structure	-16.0	-22.1	-19.2	-92.4

Emission intensity Gap	28.9	82.3	858.5	191.0
Total net emissions change (Mt)	20.49	41.61	-2.81	-17.72
**LAM**	**2004-2007**	**2007-2011**	**2011-2014**	**2014-2017**
Trade Volume	4.3	-16.1	59.6	-35.0
Trade Structure	0.1	-21.8	-220.8	12.7
Emission intensity Gap	95.6	137.9	261.2	122.3
Total net emissions change (Mt)	54.91	28.20	-1.91	-18.49
**MNA**	**2004-2007**	**2007-2011**	**2011-2014**	**2014-2017**
Trade Volume	-15.0	-20.1	-149.2	-67.9
Trade Structure	-7.0	3.3	46.8	42.1
Emission intensity Gap	122.0	116.9	202.4	125.8
Total net emissions change (Mt)	45.76	33.48	13.26	-52.31
**PAS**	**2004-2007**	**2007-2011**	**2011-2014**	**2014-2017**
Trade Volume	-16.7	-33.5	41.6	-21.4
Trade Structure	31.1	102.0	-71.4	1.1
Emission intensity Gap	85.6	31.5	129.8	120.3
Total net emissions change (Mt)	23.14	14.97	10.18	-25.10
**SSA**	**2004-2007**	**2007-2011**	**2011-2014**	**2014-2017**
Trade Volume	139.3	220.2	120.2	71.4
Trade Structure	57.3	124.0	44.5	15.8
Emission intensity Gap	-96.7	-244.2	-64.7	12.8
Total net emissions change (Mt)	15.85	-9.35	8.55	-12.93

Note: EAS: China; SAS: India; LAM: Latin America and the Caribbean; MNA: the Middle East and North Africa, PAS: Pacific Developing regions in Asia and Pacific, SSA: sub-Saharan Africa.

Table S5. Coal share and emission intensity in China, India and USA.

	2004	2007	2011	2014	2017	2018	2019
China	71.08%	73.55%	70.66%	65.77%	60.30%	58.36%	56.77%
India	51.63%	52.98%	53.30%	58.26%	55.68%	55.73%	54.44%
USA	23.26%	23.40%	21.27%	19.28%	14.92%	13.78%	11.85%
China's emission intensity (kg/$)	1.34	1.27	1.16	0.90	0.75	0.72	0.69
India's emission intensity (kg/$)	1.10	1.07	1.13	1.07	0.95	0.94	0.92
USA's emission intensity (kg/$)	0.39	0.36	0.33	0.30	0.26	0.26	0.25

*Data source: coal share and CO2 emissions data (BP Statistical Review of World Energy, 2022); GDP in constant 2015 USD price (World Bank Database).
